# The Tsunami of COVID-19 Infection Among Kidney Transplant Recipients: A Single-Center Study from Iran

**DOI:** 10.1007/s44197-021-00015-3

**Published:** 2021-11-26

**Authors:** Hormat Rahimzadeh, Seyed Saeed Tamehri Zadeh, Alireza Khajavi, Mohammad Saatchi, Leonardo Oliveira Reis, Fateme Guitynavard, Sanaz Dehghani, Venus Soleimani, Seyed Mohammad Kazem Aghamir

**Affiliations:** 1 Department of Nephrology Diseases, Sina Hospital, Tehran University of Medical Sciences, Tehran, Iran; 2 Urology Research Center, Sina Hospital, Tehran University of Medical Sciences, Tehran, Iran; 3 Student Research Committee, School of Allied Medical Sciences, Shahid Beheshti University of Medical Sciences, Tehran, Iran; 4 Research Center in Emergency and Disaster Health, University of Social Welfare and Rehabilitation Sciences (USWR), Tehran, Iran; 5 UroScience and Department of Surgery (Urology), School of Medical Sciences, University of Campinas, Unicamp and Pontifical Catholic University of Campinas, PUC-Campinas, Campinas, São Paulo, Brazil; 6 Organ Procurement Unit of Sina Hospital, Tehran University of Medical Sciences, Tehran, Iran

**Keywords:** AKI, COVID-19, Kidney, SARS-CoV-2, Transplant

## Abstract

**Background:**

Although most evidence supports the fact that kidney transplant (KT) recipients are at significant risk of morbidity and mortality, risk factors of accruing COVID-19 in this population have remained poorly defined.

**Methods:**

All KT recipients who had been transplanted in Sina Hospital and were actively followed between March 1996 and January 2021 were enrolled in a retrospective manner. The demographic characteristics, immunosuppressive treatment before KT, and death were gathered by calling patients with a designed questionnaire.

**Results:**

108 (about 21%) of 523 KT recipients were diagnosed with COVID-19. The mean age of COVID-19 patients was 46.9 ± 13.6, of whom 43% were women. In the multivariate model, body mass index (BMI) ≥ 30 independently increased the risk of COVID-19 incidence with OR 2.00 (95% CI 1.23, 3.26) (*P* = 0.00), and besides, having diabetes had a marginal association with COVID-19 incidence (OR 1.62 [95% CI 0.98, 2.66]; *P* = 0.057). The mortality rate of COVID-19 was 15%. In the multivariate model, only pre-transplantation diabetes significantly increased the risk of death by COVID-19 with OR of 3.90 (95% CI 1.00–15.16) (*P* = 0.04).

**Conclusion:**

Given the higher incidence rate in KT recipients with obesity and diabetes and higher mortality rate in KT recipients with diabetes as the cause of ESRD, more attention should be paid to KT recipients with these risk factors.

## Introduction

Currently, the entire world is tackling the novel coronavirus (SARS-CoV-2) that has caused coronavirus disease 2019 (COVID-19) [1, 2]. Scientists found that the virus can easily be transmitted through the respiratory droplets of infected patients [3]. While most patients infected with the coronavirus exhibit mild symptoms, about 20% of infected patients display severe lung injury, which is attributed to great dysregulated inflammatory responses [2, 4]. Although a bulk of evidence claims that the whole mortality rate of this virus ranges from 3.7 to 11%, several groups are at higher risk of death from the virus, particularly those with chronic underlying disease [5, 6].

Kidney transplant (KT) recipients are at higher risk of viral and bacterial infections than the general population, which primarily results from poor T-cell immunity of KT recipients [7, 8]. Moreover, it has been shown that the virus may appear in KT recipients atypically [7, 9]. To date, several studies have evaluated the impact of SARS-CoV-2 on KT recipients [10]. Pereira et al. shared their experience regarding 90 patients with solid organ transplantation in the US, among whom 46 patients had kidney transplantation. The most common manifestations were as follows: fever, cough, and dyspnea. The majority of the patients had underlying diseases that were accompanied by poor outcomes in COVID-19 patients. After a 3-week follow-up, the overall mortality reached about 18% [11]. An international survey reported 52% and 32% acute kidney injury and mortality during a median follow-up of 52 days [12].

It is generally acknowledged that there is not much reliable information concerning the incidence of COVID-19 in KT recipients. Therefore, this study attempted to evaluate the incidence of SARS-CoV-2 infection and the possible risk factors of COVID-19, acute kidney injury, and related death in all KT recipients of a single kidney transplant center in Tehran.

## Methods

### Study Population

In this study, all KT recipients who had been transplanted in Sina Hospital (Tehran, Iran) between March 1996 and January 2021 and came to the hospital transplant clinic for periodic follow-ups were included. Patients who (1) returned to dialysis due to graft loss, (2) did not seek the follow-up, and (3) died from other causes were excluded from the study. Informed consent was obtained from all participants.

### Study Design

This retrospective study was performed 1 year after the onset of the COVID-19 epidemic and at the end of its third peak in Iran (February 2021). All patients were contacted by telephone and their information was recorded in a designed questionnaire. Also, the information of a group of KT recipients, who were present in person at the time of the study or hospitalized in the center due to COVID-19 disease, was recorded. COVID-19 diagnosis was based on positive polymerase chain reaction (PCR) from nasopharyngeal, oropharyngeal swab, or abnormal chest computed tomography (CT) scan findings compatible with COVID-19 in the presence of clinical manifestations.

The designed questionnaire included questions concerning demographic characteristics including age, sex, body mass index (BMI), smoking status, opium use, existing comorbidities (diabetes, hypertension, chronic lung disease, coronary artery disease, and heart failure), Influenza vaccination before COVID-19 epidemic, the primary cause of the end-stage renal disease (ESRD) (diabetes, hypertension, kidney stone, polycystic kidney disease, other, and unknown), source of kidney graft (living or deceased), pre-transplant dialysis time (month), first or second time KT, clinical manifestations (myalgia, cough, fever, headache, shortness of breath, sore throat, phlegm, loss of smell, loss of taste, vomiting, diarrhea, and runny nose), the status of PCR test, chest CT scan involvement, immunosuppressive regimen, serum creatinine increment, admission in hospital (ICU and non-ICU setting), need to dialysis, and death.

According to the KDIGO criteria, AKI was defined as an increase in serum creatinine to ≥ 1.5 times of baseline occurring within the previous seven days or an increase in serum creatinine by ≥ 0.3 mg/dl within 48 h.

### Main Outcomes

The primary outcome was the incidence of COVID-19 disease among KT recipients in the first year of the COVID-19 epidemic in Iran. The second outcomes were as follows: (1) Factors associated with the incidence of COVID-19. (2) Incidence of AKI and mortality among KT recipients. (3) Factors associated with COVID-19 mortality.

### Statistical Analysis

Demographic characteristics of the study population were described as mean ± standard deviation (SD) for continuous variables and as frequencies (percentage). The demographic characteristics were compared using *t* test for continuous normally distributed variables, Chi-squared test for categorical variables, and Mann–Whitney *U* statistic for skewed variables. A *P* value smaller than 0.05 was significant.

To identify the factors that increase the risk of incidence and mortality of COVID-19, a simple logistic regression analysis was performed. The factors had significant associations with the incidence of COVID-19 (*P* value was set as 0.05) in the univariate model and then were included in the multivariate model. All two-tailed tests were conducted using STATA v. 14 SE (StataCorp, TX, USA).

## Results

### Patients’ Characteristics Based on COVID-19 Status

The data of 523 patients who had undergone KT and were eligible for the study were analyzed. The demographic characteristics and comorbidities of the patients based on COVID-19 status are presented in Table 1. A total of 108 patients (20.8%) were diagnosed with COVID-19; 43% were women compared with 32% without COVID-19. A significant difference was detected between the two groups regarding sex. The mean age of COVID-19 patients was 46.9 ± 13.6 years compared to 43.7 ± 13.4 years in COVID-19-negative patients. Among the assessed comorbidities, only diabetes was significantly different between the COVID-19 positive and negative patients (*P* value = 0.00). Prevalence of smoking was 7.4% in COVID-19 patients, although none had a history of opium use. Nearly half of the COVID-19 positive and negative patients had been vaccinated for Influenza before the COVID-19 epidemic and the difference of which was not significant (*P* = 0.88). The prevalence of diabetes, hypertension, renal stone, and polycystic kidney disease as primary causes of ESRD in COVID-19 patients was 13%, 37%, 3%, and 9.3%, respectively; none of them showed a significant association with COVID-19 disease. Nine percent and 7.7% of COVID-19 positive and negative patients had a history of second kidney transplantation (*P* = 0.57), respectively. The living donors accounted for 31% and 26% of COVID-19 positive and negative patients, respectively; there was no significant difference between the two groups regarding donor sources (*P* value = 0.39). The duration of pre-transplant dialysis was associated negatively with COVID-19 (*P* = 0.04).

**Table 1:** Demographic characteristics based on COVID-19 status

Demographic characteristics	COVID-19 positive patients	COVID-19 negative patients	*P* value
Women, no. (%)	46 (43)	230 (31)	0.02
Age (year)	46.9 ± 13.6	43.7 ± 13.4	0.55
BMI (kg/m^2^), no. (%)			0.00
BMI I ≥ 30	36 (33%)	77 (18%)	
BMI < 30	72 (67%)	338 (82%)	
Diabetes, no. (%)	35 (32)	82 (20)	0.00
Hypertension, no. (%)	59 (55)	230 (55)	0.88
Chronic lung disease, no. (%)	1 (1)	2 (0.5)	0.58
Coronary artery disease, no. (%)	3 (3)	6 (1)	0.34
Heart failure, no. (%)	0 (0)	2 (0.5)	0.47
Cigarette, no. (%)	8 (7)	22 (5)	0.40
Opium, no. (%)	0 (0)	13 (3)	0.08
Influenza vaccine, no. (%)	50 (47)	191 (46)	0.96
Cause of ESRD, no. (%)			
Diabetes	14 (13)	46 (11)	0.58
Hypertension	40 (37)	183 (44)	0.18
Kidney stone	3 (3)	18 (4)	0.46
Polycystic kidney disease	10 (9)	25 (6)	0.22
Other	15 (14)	79 (19)	0.21
Unknown	33(31)	92 (22.2)	0.07
Second kidney transplant, no. (%)	10 (9)	32 (7.7)	0.57
Pre-transplant dialysis time, month, no			0.18
Greater than median	48	212	
Lower than median	60	203	
Living donor, no. (%)	31 (31)	109 (26)	0.39

### Presentations of COVID-19 Patients

**Fig. 1:**
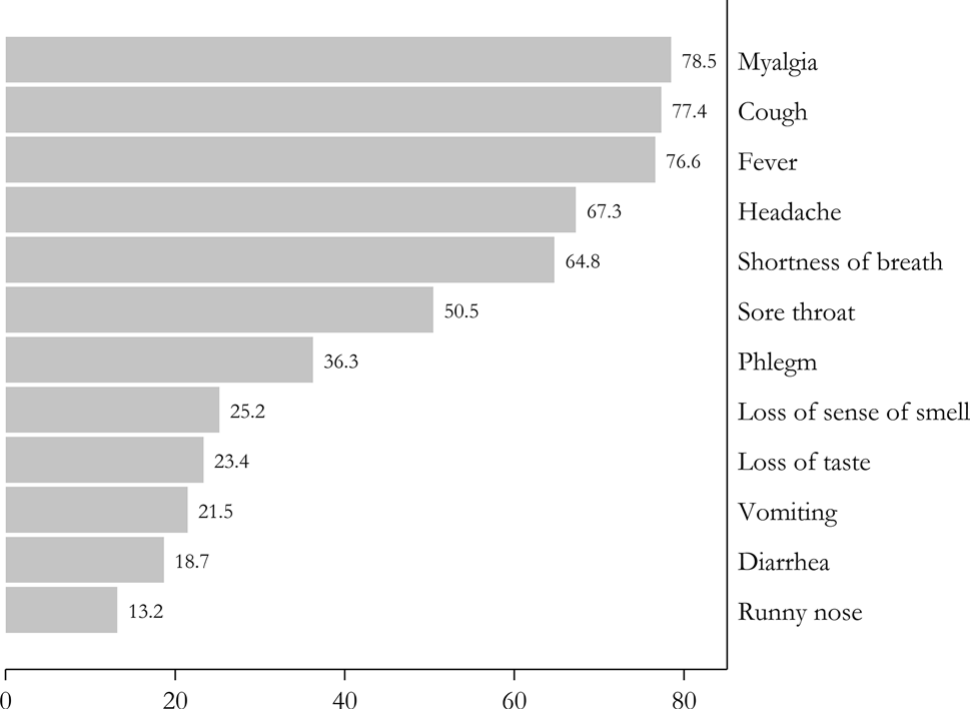
Clinical features of kidney transplant recipients with COVID-19

According to the results, the most prevalent symptom was myalgia (78.5%) followed by cough (77.4%), fever (76.6%), headache (67.3%), shortness of breath (64.8%), sore throat (50.5%), phlegm (36.3%), loss of smell (25.2%), loss of taste (23.4%) vomiting (21.5%), diarrhea (18.7%), and runny nose (13.2%) (Fig. 1). A pulmonary CT scan was performed for all the patients, and lung involvement was found in 80% of COVID-19 patients. The PCR test was positive in about 80% of COVID-19 patients as well. Forty (37%) out of 108 COVID-19 patients were recommended to stay at home, after which the questionnaire was completed via phone contact. Ten (9%) patients were transferred to the intensive-care unit (ICU) due to the need for intubation, and 58 patients (54%) were admitted to non-ICU settings. Shortness of breath, cough, and fever were the most common symptoms observed in 90% of ICU-admitted patients. Moreover, it was found out that approximately 22% of COVID-19 patients caught the virus from their own families, because at least one of their family members had recently developed COVID-19.

### COVID-19-Related AKI

AKI was detected in 28 (26%) patients, four of whom expired. There was no significant association between AKI and death (*P* = 0.56). During the study, dialysis was performed for three COVID 19 patients with AKI. Of these patients, one died, and one patient rejected the transplanted kidney and was back on hemodialysis. No significant association was found between performing dialysis and death (*P* = 0.36).

### Risk Factors of Incidence of COVID-19

In the crude model, sex, diabetes, BMI, and pre-transplant dialysis duration had a significant association with COVID-19. BMI ≥ 30 and pre-transplant dialysis duration (per1 month increment) independently increased and decreased the risk of COVID-19 with OR of 2.00 (1.23, 3.26) (*P* = 0.005) and 0.98 (0.97, 0.99) (*P* = 0.044), respectively. Additionally, having diabetes marginally increased the risk of COVID-19 incidence with an OR of 1.62 (0.98, 2.66) (*P* = 0.057) (Table 2).

**Table 2:** Multivariate odds ratio (OR) and 95% confidence interval (CI) of incidence of COVID-19 disease among kidney transplant recipients

Factors associated with incidence of COVID-19 disease	OR (95% CI)	*P* value
Diabetes, yes	1.62 (0.98, 2.66)	0.05
Sex (female as reference)	1.46 (0.93, 2.30)	0.09
BMI, kg/m^2^ (BMI ≥ 30)	2.00 (1.23, 3.26)	0.00
Pre-transplant dialysis time per 1-month increment	0.98 (0.97, 0.99)	0.04

### COVID-19-Related Death

Concerning mortality, 16 (15%) of COVID-19-positive patients died because of COVID-19, half of whom had been admitted to ICU and half to non-ICU settings. The mortality rate was significantly higher for ICU patients than non-ICU with an OR of 25 [95% CI 4.42–141.39]. In the crude model, old patients, smokers, and patients with pre-transplantation diabetes and hypertension were more likely to die from COVID-19 (Table 3). In the multivariate model, only pre-transplantation diabetes increased the risk of COVID-19 death with an OR of 3.90 [95% CI 1.00–15.16] (*P* = 0.04) (Table 4).

**Table 3:** Baseline demographics based on COVID-19 mortality status

Demographic characteristics and comorbidities	Non-survivor patients	Survivor patients	*P* value
Men, no. (%)	9 (56)	53 (58)	0.91
Age (year)	56.13 ± 11.28	45.46 ± 13.44	0.00
BMI (kg/m^2^)			0.54
BMI I ≥ 30	4	30	
BMI < 30	12	62	
Diabetes, no. (%)	8 (50)	27 (29)	0.10
Hypertension, no. (%)	14 (87)	45 (92)	0.00
Chronic lung disease, no. (%)	0 (0)	1 (1)	1.00
Coronary artery disease, no. (%)	0 (0)	3 (3)	1.00
Heart failure, no. (%)	0 (0)	0 (0)	–
Cancer, no. (%)	0 (0)	1 (1)	1.00
Cigarette, no. (%)	4 (25)	4 (4)	0.00
Opium, no. (%)	0 (0)	0 (0)	–
Influenza vaccine, no. (%)	4 (25)	46 (50)	0.06
Cause of ESRD, no. (%)			
Diabetes	6 (37)	8 (11)	0.00
Hypertension	10 (62)	30 (32)	0.02
Kidney stone	0 (0)	3 (3)	1.00
Polycystic kidney disease	0 (0)	10 (11)	0.35
Other	0 (0)	15 (16)	0.12
Unknown	3(19)	30 (33)	0.38
Second kidney transplant, no. (%)	1 (6)	9 (10)	1.00
Living donor, no. (%)	3 (19)	30 (33)	0.38
Time from transplant to COVID-19 symptoms onset, no. (%)			0.12
> 1 year	13 (81)	86 (93)	
< 1 year	3 (19)	6 (7)

**Table 4:** Multivariate odds ratio (OR) and 95% confidence interval (CI) of death of COVID-19 disease among kidney transplant recipients

Factors associated with death of COVID-19 disease	OR (95% CI)	*P* value
Age, years	1.04 (0.99, 1.09)	0.07
Smoking, yes	4.48 (0.65, 30.83)	0.12
Pre-transplantation diabetes, yes	3.90 (1.00, 15.16)	0.04
Pre-transplantation hypertension, yes	3.22 (0.86, 12.02)	0.08

### Association Between Immunosuppressive Drugs and COVID-19 Incidence and Death

COVID-19-positive patients were on prednisone (97%), Mycophenolate Mofetil (61%), Cyclosporine (48%), Tacrolimus (45%), Mycophenolate Sodium (26%), Sirolimus (7%), and Azathioprine (6%). None of these drugs showed a significant association with COVID-19 status and death (all *P* values > 0.05). At least one of Mycophenolate Mofetil, Mycophenolate Sodium, and Azathioprine, known as antimetabolites drugs was used by 101 (93%) and 393 (95%) COVID-19 positive and negative patients, respectively. No significant difference was observed between the two groups regarding antimetabolites drugs usage (*P* = 0.63).

## Discussion

To the best of our knowledge, the current study is the second study to date assessing the incidence and risk factors of COVID-19 and its related death in all KT recipients, as one of the most susceptible groups to death from COVID-19, followed by a single center. The study demonstrated that the incidence of COVID-19 among Iranian KT recipients is approximately 21%, among whom 15% expired owing to COVID-19. Furthermore, diabetes, obesity, and shorter dialysis duration prior to transplantation acted as independent risk factors for the incidence of COVID-19.

In theory, all solid organ transplant recipients, particularly KT recipients, are more predisposed to infections like SARS-CoV-2 than the general population, which mainly originated from prolonged use of immunosuppression drugs [12]. In practice, the incidence of COVID-19 in KT recipients has by far been investigated in few studies. In Elias et al., 66 (5%) out of 1216 KT recipients were diagnosed with COVID-19 during an 8-week follow-up in two transplant centers in Paris, in comparison with France's general population (0.3%), was remarkably higher [13]. Another study claimed that while 16.6% of KT recipients were positive for SARS-CoV-2 IgG antibody, nearly one-third of whom had positive PCR test for SARS-CoV-2, and the rest had no clinical symptoms evaluated for COVID-19. They also pointed out that the prevalence of COVID-19 in their center based on antibody and PCR test was 23.4% during the median follow-up of 44 days [14]. The incidence of COVID-19 in our study (21%) was close to the study of Azzi et al. and remarkably higher than the study by Elias et al. Notably, due to different times of peak of COVID-19 in different regions, the incidence of the disease is not comparable between different regions. As of January 31, 2021, 1,417,999 confirmed cases of COVID-19 were reported in Iran. Thus, the incidence rate of COVID-19 in Iran is about 2% (according to the total population of around 80 million in 2016 [15]). Therefore, as expected, KT recipients are at a higher risk of catching COVID-19 than the general population in Iran. It is of crucial importance to note that the prevalence of COVID-19 in the present study was underestimated, because there could be some KT recipients infected with COVID-19 without any clinical symptoms, since COVID-19 diagnosis was based on clinical symptoms with either positive PCR or positive CT scan.

Up to now, only Elias et al. have assessed the factors associated with the incidence of COVID-19 in KT recipients. They pointed out that non-white ethnicity, obesity, asthma, and chronic pulmonary disease, and diabetes are capable of significantly increasing the risk of incidence of COVID-19 with ORs of 2.17 [95% CI 1.23–3.78], 2.19 [95% CI 1.19–4.05], 3.09 [95% CI 1.49–6.41], and 3.33 [95% CI 1.92–5.77] [13]. In the study by Elhadedy et al., hypertension and diabetes were the most prevalent comorbidities among KT recipients with COVID-19 [16]. Likely, in the study by Maheboob et al., diabetes, hypertension, and chronic pulmonary disease were the most observed comorbidities [17]. The present study found that diabetes, obesity (as shown by BMI > 30), and shorter pre-transplant dialysis period independently increased the risk of COVID-19 among KT recipients. Hence, concerning being infected with COVID-19, KT recipients with diabetes and obesity should observe more precautions than others. No justification could be offered for the association between shorter dialysis duration and higher COVID-19 incidence, which is suggested to be further evaluated in future studies.

KT recipients are more likely to be at higher risk of developing complications of COVID-19 than the general population, not only due to their chronic use of immunosuppressive drugs but also because of having serious comorbidities, such as diabetes and hypertension, and cardiovascular disease [11, 18, 19]. In the current study, the mortality rates among the whole, non-ICU hospitalized, and ICU patients were 15%, 14%, and 80%, respectively. Cravedi et al. reported an overall mortality rate of 32%, and 51% of ICU-admitted patients died [12]. Pereira et al. reported that during 3-week follow-up, 18%, 24%, and 52% of the whole hospitalized and ICU-admitted patients passed away, respectively [11]. In a study by Nikpouraghdam, Iran's overall and hospitalized mortality rates reached 1.85% and 8.06% [20], which are remarkably lower than KT recipients. Furthermore, a meta-analysis demonstrated that ICU patients are at 2.72-fold higher risk of death from COVID-19 compared to non-ICU patients [21]; however, this ratio for our patients reached 6. They also declared that 18.8% of COVID-19 hospitalized patients expired [21]; nevertheless, this value was 23% in the current study. Therefore, KT recipients as highly vulnerable patients should receive more attention.

Several studies have been conducted on predictors of mortality among KT recipients [22]. In the multivariate model, diabetes as the primary cause of ESRD in this study had a significant association with higher mortality risk. Azzi et al. indicated that diabetes as the primary cause of ESRD demonstrated a significant association with mortality in the univariate model but not in the multivariate model [14]. It has been shown that among the KT candidates, diabetes as a primary diagnosis enhanced the mortality rate with the incidence rate of 2.07 [95% CI 1.43, 3.00] [23]. An observational study revealed that age > 60 years, cardiovascular disease, and dyspnea independently increased mortality risk among KT recipients [24]. A very recent meta-analysis showed that obesity significantly enhances hospital mortality [25]. Likely, a multi-center cohort study found obesity as an independent risk factor for mortality in solid organ transplant recipients with OR of 1.9 (95% CI 1.4, 7) [26]. On the other hand, a French cohort did not find any significant association between mortality and BMI > 25 in KT recipients [24].

AKI is one of the most common complications appearing after COVID-19 in KT recipients and is observed in 30–89% of hospitalized KT recipients. This value was 26% in our study. The probable mechanisms of AKI are a decrease in renal perfusion, cytokine storm syndrome, rhabdomyolysis, hypoxia, and dehydration [22, 27]. The virus can also prompt tubular damage through the infiltration of macrophages, mostly CD68+, and tubular deposition of C5b-9 [25]. Several risk factors have been proposed for AKI in KT recipients. A recent meta-analysis declared that age but not sex, time after transplantation, and comorbidities, acts as a risk factor for AKI, and studies with a mean age of > 60 years had the highest incidence of AKI, while studies with a mean age of < 50 years had the lowest incidence of AKI [28]. Furthermore, we did not detect higher risk of mortality in patients who experienced AKI. A multi-center cohort study from India found AKI in 121(48.4%) KT recipients, of whom 93 survived and achieved a significant association between AKI and mortality [29]. It is not often permanent; however, some studies reported graft loss following AKI ranging from 4 to 11% [24, 30]. In the current study, among AKI patients, one patient (nearly 4%) experienced graft loss.

About one-fifth of our patients with COVID 19 had caught the disease from a family member. Nair et al. showed that three (30%) of ten KT recipients with COVID-19 were infected with SARS-CoV-2 from their family members [7]. Since family members can be a major source of COVID-19 for patients, it has been suggested that KT recipients keep their distance from their own families, as well. Since family members can be a major source of COVID-19 for patients, it has been suggested that KT recipients keep their distance from their own families.

This is the second study assessing COVID-19 incidence and related risk factors among KT recipients [31]. Another strength of the study is its large population. It also evaluates all KT recipients of a medical center during a whole year, whereas Elias et al. assessed patients for about 2 months. However, there are some limitations to be acknowledged as well.

1. The nature of the study is retrospective; hence, long-term prospective studies are necessary to confirm the findings.
2. The incidence rate of COVID-19 between KT recipients is affected by factors like isolation and precautions; therefore, it is difficult to prove a direct correlation between patients' comorbidities and the incidence of COVID-19.
3. Since phone interviews and questionnaires gathered the data, there was no access to information such as some laboratory results and applied treatment strategies for patients admitted to other hospitals.
4. The lack of PCR tests in a part of the study population was another limitation.
5. Because the study was conducted in a single center in Tehran, there is a need for multi-center studies with different ethnicities.

## Conclusions

We demonstrated that the incidence of COVID-19 among KT recipients is much higher than in the general population. Furthermore, we observed that diabetes, obesity, and shorter pre-transplant dialysis time independently increased the risk of COVID-19. Concerning the death rate among KT recipients, diabetes as the cause of ESRD independently enhanced the risk of death by COVID-19.

## Data Availability

Data will be available on request.

## Abbreviations

KT: Kidney transplant
COVID-19: Coronavirus disease 2019
SARS-CoV-2: Severe acute respiratory syndrome coronavirus 2
PCR: Polymerase chain reaction
CT scan: Computed tomography scan
BMI: Body mass index
ESRD: End-stage renal disease
AKI: Acute kidney injury
ICU: Intensive-care unit
KDIGO: Kidney disease: improving global outcomes
OR: Odds ratio

## References

[1] Khatami F (2020). A meta-analysis of accuracy and sensitivity of chest CT and RT-PCR in COVID-19 diagnosis. Sci Rep.

[2] Yang X (2020). Clinical course and outcomes of critically ill patients with SARS-CoV-2 pneumonia in Wuhan, China: a single-centered, retrospective, observational study. Lancet Respir Med.

[3] Bai Y (2020). Presumed asymptomatic carrier transmission of COVID-19. JAMA.

[4] Zhou M, Zhang X, Qu J (2020). Coronavirus disease 2019 (COVID-19): a clinical update. Front Med.

[5] Cao Y (2020). Imaging and clinical features of patients with 2019 novel coronavirus SARS-CoV-2: a systematic review and meta-analysis. J Med Virol.

[6] Cheng Y (2020). Kidney disease is associated with in-hospital death of patients with COVID-19. Kidney Int.

[7] Nair V (2020). COVID-19 in kidney transplant recipients. Am J Transplant.

[8] Vistoli F (2020). COVID-19 and kidney transplantation: an Italian Survey and Consensus. J Nephrol.

[9] Godbole G, Gant V (2013). Respiratory tract infections in the immunocompromised. Curr Opin Pulm Med.

[10] Mahalingasivam, V., et al., COVID-19 and kidney transplantation: A systematic review. Kidney international reports, 2020. 10.1016/j.ekir.2020.10.023 PMC7607258 33163708

[11] Pereira MR (2020). COVID-19 in solid organ transplant recipients: initial report from the US epicenter. Am J Transplant.

[12] Cravedi P (2020). COVID-19 and kidney transplantation: results from the TANGO International Transplant Consortium. Am J Transplant.

[13] Elias M (2020). COVID-19 infection in kidney transplant recipients: disease incidence and clinical outcomes. J Am Soc Nephrol.

[14] Azzi Y (2020). COVID-19 infection in kidney transplant recipients at the epicenter of pandemics. Kidney Int.

[15] Danaei G (2019). Iran in transition. Lancet.

[16] Elhadedy MA, Marie Y, Halawa A (2021). COVID-19 in renal transplant recipients: case series and a brief review of current evidence. Nephron.

[17] Maheboob S et al. Clinical outcomes of post renal transplant patients with COVID-19 infection: a single-center case series. Authorea Preprints, 2021. 10.1002/ccr3.4513 PMC8299096 34322259

[18] Akalin E (2020). Covid-19 and kidney transplantation. N Engl J Med.

[19] Kataria A (2020). COVID-19 in kidney transplantation: epidemiology, management considerations, and the impact on kidney transplant practice. Transplant Direct.

[20] Nikpouraghdam M (2020). Epidemiological characteristics of coronavirus disease 2019 (COVID-19) patients in IRAN: a single center study. J Clin Virol.

[21] Noor FM, Islam MM (2020). Prevalence and associated risk factors of mortality among COVID-19 patients: a meta-analysis. J Community Health.

[22] Azzi Y (2021). Covid-19 and solid organ transplantation: a review article. Transplantation.

[23] Schold JD (2021). COVID-19 mortality among kidney transplant candidates is strongly associated with Social Determinants of Health. Am J Transplant.

[24] Caillard S (2020). An initial report from the French SOT COVID Registry suggests high mortality due to Covid-19 in recipients of kidney transplants. Kidney Int.

[25] Yang J (2021). Obesity aggravates COVID-19: an updated systematic review and meta-analysis. J Med Virol.

[26] Kates OS et al. COVID-19 in solid organ transplant: a multi-center cohort study. Clin Infect Dis. 2020.

[27] Rahimzadeh H (2021). COVID-19 progression in kidney transplant recipients: a single-center case series. CEN Case Rep.

[28] Kremer D et al. A systematic review and meta‐analysis of COVID‐19 in kidney transplant recipients: lessons to be learned. Am J Transplant. 2021. 10.1111/ajt.16742 PMC9292797 34212499

[29] Kute VB (2021). Clinical profile and outcome of COVID-19 in 250 kidney transplant recipients: a multi-center cohort study from India. Transplantation.

[30] Lubetzky M (2020). Kidney allograft recipients, immunosuppression, and coronavirus disease-2019: a report of consecutive cases from a New York City transplant center. Nephrol Dial Transplant.

[31] Marinaki S (2020). A systematic review of COVID-19 infection in kidney transplant recipients: a universal effort to preserve patients’ lives and allografts. J Clin Med.

